# Efficacy and safety of biosimilar CT-P17 versus reference adalimumab in subjects with rheumatoid arthritis: 24-week results from a randomized study

**DOI:** 10.1186/s13075-020-02394-7

**Published:** 2021-02-05

**Authors:** Jonathan Kay, Janusz Jaworski, Rafal Wojciechowski, Piotr Wiland, Anna Dudek, Marek Krogulec, Slawomir Jeka, Agnieszka Zielinska, Jakub Trefler, Katarzyna Bartnicka-Maslowska, Magdalena Krajewska-Wlodarczyk, Piotr A. Klimiuk, Sang Joon Lee, Yun Ju Bae, Go Eun Yang, Jae Kyoung Yoo, Daniel E. Furst, Edward Keystone

**Affiliations:** 1 University of Massachusetts Medical School and UMass Memorial Medical, Worcester, MA USA; 2 Reumatika-Centrum Reumatologii, Warsaw, Poland; 3 University Hospital No 2, Bydgoszcz, Poland; 4 Medical University, Wroclaw, Poland; 5 Centrum Medyczne AMED, Warsaw, Poland; 6 Rheumatology Clinic NZOZ Lecznica MAK-MED, Nadarzyn, Poland; 7 Nasz Lekarz Przychodnie Medyczne, Toruń, Poland; 8 Medycyna Kliniczna Marzena Waszczak-Jeka, Warsaw, Poland; 9 Reuma Centrum, Warsaw, Poland; 10 Centrum Medyczne AMED Oddzial w Lodzi, Łódź, Poland; 11 University of Warmia and Mazury, Olsztyn, Poland; 12 Medical University of Bialystok and Gabinet Internistyczno-Reumatologiczny Piotr Adrian Klimiuk, Białystok, Poland; 13 Celltrion, Inc., Incheon, Republic of Korea; 14 University of California, Los Angeles, CA USA; 15 University of Washington, Seattle, WA USA; 16 University of Florence, Florence, Italy; 17 University of Toronto, Toronto, Canada

**Keywords:** Adalimumab, Biologics, Biosimilars, Comparative effectiveness, Immunogenicity, Monoclonal antibodies, Rheumatoid arthritis, Safety, Tumor necrosis factor inhibitors

## Abstract

**Background:**

To demonstrate equivalent efficacy of the proposed high-concentration (100 mg/ml), citrate-free adalimumab biosimilar CT-P17 to European Union-approved adalimumab (EU-adalimumab) in subjects with active rheumatoid arthritis (RA).

**Methods:**

This randomized, double-blind phase III study (ClinicalTrials.gov, NCT03789292) randomized (1:1) subjects with active RA at 52 centers to receive CT-P17 or EU-adalimumab 40 mg subcutaneously every 2 weeks until week 52. Results to week 24 are reported here. The primary endpoint was 20% improvement by American College of Rheumatology criteria (ACR20) response rate at week 24. Equivalence was concluded if the corresponding confidence intervals (CIs) for the estimate of treatment difference were within predefined equivalence margins: − 15 to 15% (95% CI; European Medicines Agency assumption); − 12 to 15% (90% CI; Food and Drug Administration assumption). Additional efficacy, pharmacokinetic, usability, safety, and immunogenicity endpoints were evaluated.

**Results:**

648 subjects were randomized (324 CT-P17; 324 EU-adalimumab). The ACR20 response rate at week 24 was 82.7% (*n* = 268/324) in both groups (intention-to-treat population). The 95% CI (− 5.94 to 5.94) and 90% CI (− 4.98 to 4.98) were within predefined equivalence margins for both assumptions and equivalent efficacy was concluded. Additional endpoints and overall safety were comparable between groups. Mean trough serum concentrations of CT-P17 were slightly higher than those of EU-adalimumab. Immunogenicity was slightly lower numerically for the CT-P17 group than for the EU-adalimumab group.

**Conclusions:**

CT-P17 and EU-adalimumab have equivalent efficacy and comparable safety and immunogenicity in subjects with active RA. Overall safety of CT-P17 is consistent with the known safety profile of reference adalimumab.

**Trial registration:**

ClinicalTrials.gov, NCT03789292. Registered 28 December 2018—retrospectively registered.

## Background

Biological disease-modifying antirheumatic drugs (bDMARDs), such as tumor necrosis factor (TNF) inhibitors, are recommended for the treatment of rheumatoid arthritis (RA) when disease activity remains moderate or high despite conventional synthetic DMARD (csDMARD) monotherapy [1]. Adalimumab is an anti-TNF monoclonal antibody that effectively treats RA [2–5]. Biosimilars are highly similar to their reference products in terms of quality characteristics, biological activity, safety, and efficacy [6]. Since 2016, several adalimumab biosimilars have been licensed by the US Food and Drug Administration (FDA) and the European Medicines Agency (EMA) [7, 8]. European League Against Rheumatism (EULAR) recommendations for the treatment of RA position biosimilar DMARDs (bsDMARDs) equivalently to their reference products in treatment algorithms, and suggest that lower-priced biosimilars are preferred for their potential to reduce healthcare expenditures [9].

CT-P17 is in development as a proposed adalimumab biosimilar [10, 11]. CT-P17 is administered at 100 mg/ml, reflecting the high-concentration formulation of reference adalimumab [12–14], and is also citrate-free, which could reduce discomfort during injection [12, 15]. To date, CT-P17 has been evaluated in two randomized phase I studies evaluating the pharmacokinetics (PK) and safety of CT-P17 in healthy adults: a double-blind study comparing CT-P17 to European Union-approved adalimumab (EU-adalimumab) and US-licensed adalimumab (ClinicalTrials.gov NCT03970824) [11] and an open-label study comparing CT-P17 administration via autoinjector or prefilled syringe (ClinicalTrials.gov NCT04295356) [16].

This randomized, active-controlled, double-blind, multicenter, phase III study was designed to demonstrate that the efficacy of CT-P17 at week 24 is equivalent to that of EU-adalimumab. The study also evaluated PK, usability, and overall safety, including immunogenicity.

## Methods

### Study design and procedures

This randomized, double-blind, active-controlled, multicenter, phase III study was conducted at 52 centers in six countries (Bulgaria, Hungary, Lithuania, Peru, Poland, Ukraine; see Supplementary Table 1, Additional file 1). There were three study periods: screening (days − 42 to − 1), treatment (weeks 0–48), and end-of-study (week 52). Before dosing at week 0, subjects were randomized (1:1) to receive treatment with 40 mg (100 mg/ml) of either CT-P17 or EU-adalimumab (Humira, AbbVie Deutschland GmbH Co. KG, Ludwigshafen, Germany) every 2 weeks (q2w) until week 24 (treatment period 1). Before dosing at week 26, subjects in the EU-adalimumab group were randomized (1:1) either to continue EU-adalimumab or to switch to CT-P17 (both q2w until week 48) (treatment period 2). Subjects receiving CT-P17 during treatment period 1 continued to receive CT-P17 in treatment period 2. Results up to week 24 are reported here.

CT-P17 and EU-adalimumab were administered by subcutaneous injection via prefilled syringe. After training in proper injection technique, subjects (or caregivers, as needed) could self-administer injections at home, unless injection at the study center was required for usability assessment. Subjects also received treatment with methotrexate (MTX; 12.5–25 mg/week, or 10 mg/week if intolerant to a higher dose, oral or parenteral [intramuscular or subcutaneous] dose) and folic acid (≥ 5 mg/week, oral).

Randomization was conducted using an interactive web response system (IWRS). The biostatistics team used Rave Randomization and Trial Supply Management (Medidata Solutions, New York) to generate the randomization schedule for the IWRS, which linked sequential subject randomization numbers to treatment codes. Randomization was by permuted block (block size remains blinded until final database lock) and was stratified by country and Simplified Disease Activity Index (SDAI) at screening (> 26 vs ≤ 26). As prespecified in the protocol, the study was unblinded for reporting purposes after the database lock for data up to week 24. Efficacy, PK, usability, immunogenicity, and safety endpoints were evaluated by separate, predefined unblinded teams constituted by the sponsor and by the Contract Research Organization (CRO). The investigators, subjects, and other teams in the CRO and the sponsor will remain blinded until the end of the study.

The study was performed in accordance with the Declaration of Helsinki [17] and Good Clinical Practice guidelines [18]. All national, state, and local laws or regulations were followed. Before study initiation, the study protocol was reviewed and approved by the independent ethics committee/institutional review board at each site (see Supplementary Table 1, Additional file 1). All subjects provided written informed consent. The study was registered with ClinicalTrials.gov (NCT03789292).

### Subjects

Full eligibility criteria are detailed in Additional file 1. Subjects ranged between 18 and 75 years of age, were diagnosed with RA according to the 2010 American College of Rheumatology (ACR)/EULAR classification criteria [19], and had active disease, defined by the presence of ≥ 6 swollen joints (of 66 assessed), ≥ 6 tender joints (of 68 assessed), and either erythrocyte sedimentation rate (ESR) > 28 mm/hour or serum C-reactive protein (CRP) concentration > 1.0 mg/dl (> 10 mg/l) at screening. Subjects had received oral or parenteral MTX at a dose of 12.5–25 mg/week, or 10 mg/week if intolerant to a higher dose, for ≥ 12 weeks, and had been on a stable dose and route of administration of MTX for ≥ 4 weeks before the first administration of study drug (day 1). Key exclusion criteria included prior bDMARD or targeted synthetic DMARD treatment for RA or prior TNF inhibitor treatment for any diagnosis; active or latent tuberculosis, or history of tuberculosis; or history of or current serious infection.

### Study endpoints

The primary efficacy endpoint was the proportion of subjects achieving clinical response according to 20% improvement by ACR response criteria (ACR20) at week 24. Secondary efficacy endpoints up to week 24 were ACR20, ACR50, and ACR70 response, hybrid ACR response, Disease Activity Score in 28 joints (DAS28)-CRP response, EULAR (CRP) response, SDAI and Clinical Disease Activity Index (CDAI) remission rate, and 36-item Short Form Health Survey (SF-36) physical and mental component scores. DAS28-CRP and Boolean remission rates were analyzed post hoc. Trough serum adalimumab concentration (*C*_trough_) was evaluated as a secondary PK endpoint. Usability evaluations (Bulgaria and Poland only) included subject-reported outcomes from the Self-Injection Assessment Questionnaire (SIAQ) administered before (PRE-SIAQ) and after (POST-SIAQ) self-injection, and successful self-injection as determined by Self-Injection Assessment Checklist completed by study center staff. Safety was evaluated throughout; immunogenicity and local site pain were also assessed.

### Study assessments

Study assessments and time points for evaluation are specified in Supplementary Table 2 (Additional file 1). For efficacy assessments, procedures were performed at the study center before study drug administration. A blinded independent joint assessor was assigned at each study center. Blood samples for PK analysis were obtained predose (immediately before study drug injection) for all PK sampling time points. Usability assessments were conducted at weeks 4, 6, 8, and 24. Safety assessments performed throughout included treatment-emergent adverse events (TEAEs), TEAEs of special interest (TEAESI), immunogenicity, clinical monitoring for tuberculosis, and review of prior and concomitant medications. TEAEs were recorded according to the Common Terminology Criteria for Adverse Events v5.0 and were coded to System Organ Class (SOC) and Preferred Term according to the Medical Dictionary for Regulatory Affairs v22.0. Prior and concomitant medications were coded using the World Health Organization Drug Dictionary (March 2019 version). Protocol-specified TEAESIs were injection-site reactions (ISRs), hypersensitivity/allergic reactions, infections, and malignancies. Local site pain was assessed by using a 100-mm visual analog scale (VAS) at all study visits (except weeks 12 and 20).

Immunogenicity was evaluated at all study visits. Anti-drug antibodies (ADAs) were detected using a validated electrochemiluminescent bridging assay with acid dissociation. ADA-positive samples underwent further analysis to confirm the specificity of binding and to quantify ADA titer. If a sample was confirmed positive for specific ADAs, the presence of neutralizing antibodies (NAbs) was investigated. A validated electrochemiluminescent assay with affinity capture elution was used to measure neutralizing activity against adalimumab in human serum.

### Statistical analyses

A sample size of 450 subjects (225 per treatment group) was determined to provide ≥ 80% statistical power to demonstrate equivalence of ACR20 response at week 24, using nQuery Adviser (v7.0; nQuery, Boston, MA). This calculation was based on two sets of statistical assumptions to meet the different requirements of regulatory authorities in the European Union and the USA: an equivalence margin of − 15 to 15% using a 2 1-sided 2.5% significance level of an equivalence test (predefined in the protocol; EMA assumption), and an asymmetric equivalence margin of − 12 to 15% using a 2 1-sided 5% significance level of an equivalence test (FDA assumption). To allow for a possible dropout rate of 20%, the target sample size was 564 subjects (282 per treatment group).

Analysis populations are described in Additional file 1. The intention-to-treat (ITT) population included all subjects enrolled and randomized to receive a dose of either study drug, regardless of whether study drug dosing was completed. The ITT population was the primary analysis population for the primary endpoint, which was also assessed in the per-protocol (PP) population as a supportive analysis. The analysis was conducted by the exact binomial approach using a Farrington-Manning score method (inverting 2 1-sided test) [20]. A sensitivity analysis for the primary efficacy endpoint was performed in both the ITT and the PP populations using logistic regression with treatment group as a fixed effect and country and disease activity by SDAI at screening as covariates. Selected analyses were also conducted by ADA status. Post hoc analyses were conducted to compare parameters between treatment groups (Tables 1, 2, and 3), with *p* values generated by the Wald test (for proportional values) or *t* test (for mean values)*.* All statistical analyses were performed using SAS software v9.4 (SAS Institute, Cary, NC).

**Table 1:** Demographics and baseline disease characteristics (ITT population, unless otherwise specified)

	CT-P17 (*N* = 324)	EU-adalimumab (*N* = 324)
Age (years), median (range)	53.5 (18–75)	54.0 (19–75)
Sex, *n* (%)		
Male	75 (23.1)	59 (18.2)
Female	249 (76.9)	265 (81.8)
Race, *n* (%)		
White	299 (92.3)	298 (92.0)
Mestizo	24 (7.4)	26 (8.0)
Native Peruvian	1 (0.3)	0
Ethnicity, *n* (%)		
Hispanic or Latino	29 (9.0)	34 (10.5)
Non-Hispanic or non-Latino	295 (91.0)	290 (89.5)
RA disease duration (years), mean (SD)	6.79 (6.76)	6.59 (6.81)
SDAI at screening, *n* (%)		
SDAI ≤ 26	30 (9.3)	34 (10.5)
SDAI > 26	294 (90.7)	290 (89.5)
SDAI, mean (SD)	40.0 (11.5)	39.8 (11.1)
CDAI, mean (SD)	39.0 (11.0)	38.7 (10.8)
DAS28-CRP, mean (SD)	5.538 (0.8738)	5.547 (0.8525)
Tender joint count, mean (SD)	20.5 (10.2)	20.1 (10.1)
Swollen joint count, mean (SD)	14.0 (6.33)	14.0 (6.46)
Subject’s assessment of pain, mean (SD)^a^	69.7 (18.7)	70.0 (16.2)
Subject’s global assessment of disease activity, mean (SD)^a^	69.8 (17.8)	69.6 (16.3)
Physician’s global assessment of disease activity, mean (SD)^a^	67.5 (14.7)	67.0 (15.5)
HAQ estimate of physical ability, mean (SD)	1.41 (0.59)	1.48 (0.56)
CRP (mg/dl), mean (SD)	0.975 (1.60)	1.10 (1.91)
ESR (mm/h), mean (SD)	42.3 (15.98)	42.9 (16.94)

Note: There were no significant differences between the CT-P17 and EU-adalimumab groups for any parameter (*p* > 0.05). For age (years), mean (SD) values were used for the statistical analysis

^a^Assessed by 100-mm visual analog scale

*Anti-CCP* anti-cyclic citrullinated peptide, *CDAI* Clinical Disease Activity Index, *CRP* C-reactive protein, *DAS28* Disease Activity Score in 28 joints, *ESR* erythrocyte sedimentation rate, *EU-adalimumab* European Union-approved adalimumab, *HAQ* Health Assessment Questionnaire, *ITT* intention-to-treat, *RA* rheumatoid arthritis, *RF* rheumatoid factor, *SD* standard deviation, *SDAI* Simplified Disease Activity Index

**Table 2:** EULAR (CRP) response rate and SDAI, CDAI, DAS28 (CRP), and Boolean remission rates up to week 24 (ITT population)

	CT-P17 (*N* = 324)	EU-adalimumab (*N* = 324)
EULAR (CRP) response, *n* (%)		
Week 2		
Good response	22 (6.8)	13 (4.0)
Moderate response	154 (47.5)	139 (42.9)
Week 4		
Good response	72 (22.2)	68 (21.0)
Moderate response	162 (50.0)	167 (51.5)
Week 8		
Good response	133 (41.0)	123 (38.0)
Moderate response	143 (44.1)	144 (44.4)
Week 12		
Good response	162 (50.0)	165 (50.9)
Moderate response	131 (40.4)	124 (38.3)
Week 16		
Good response	181 (55.9)	174 (53.7)
Moderate response	112 (34.6)	109 (33.6)
Week 20		
Good response	202 (62.3)	201 (62.0)
Moderate response	91 (28.1)	87 (26.9)
Week 24		
Good response	208 (64.2)	208 (64.2)
Moderate response	86 (26.5)	81 (25.0)
CDAI remission rate, *n* (%)		
Week 2	2 (0.6)	2 (0.6)
Week 4	11 (3.4)	11 (3.4)
Week 8	21 (6.5)	24 (7.4)
Week 12	47 (14.5)	42 (13.0)
Week 16	51 (15.7)	63 (19.4)
Week 20	69 (21.3)	85 (26.2)
Week 24	82 (25.3)	86 (26.5)
SDAI remission rate, *n* (%)		
Week 2	2 (0.6)	2 (0.6)
Week 4	12 (3.7)	12 (3.7)
Week 8	22 (6.8)	22 (6.8)
Week 12	47 (14.5)	44 (13.6)
Week 16	53 (16.4)	65 (20.1)
Week 20	69 (21.3)	87 (26.9)
Week 24	86 (26.5)	93 (28.7)
DAS28 (CRP) remission rate, *n* (%)		
Week 2	11 (3.4)	10 (3.1)
Week 4	32 (9.9)	38 (11.7)
Week 8	67 (20.7)	68 (21.0)
Week 12	109 (33.6)	107 (33.0)
Week 16	128 (39.5)	118 (36.4)
Week 20	150 (46.3)	146 (45.1)
Week 24	158 (48.8)	157 (48.5)
Boolean remission rate, *n* (%)		
Week 2	2 (0.6)	2 (0.6)
Week 4	9 (2.8)	10 (3.1)
Week 8	17 (5.2)	17 (5.2)
Week 12	32 (9.9)	33 (10.2)
Week 16	40 (12.3)	56 (17.3)
Week 20	56 (17.3)	66 (20.4)
Week 24	58 (17.9)	68 (21.0)

Note: There were no significant differences between the CT-P17 and EU-adalimumab groups for any parameter (*p* > 0.05)

*CDAI* Clinical Disease Activity Index, *CRP* C-reactive protein, *DAS28* Disease Activity Score in 28 joints, *EU-adalimumab* European Union-approved adalimumab, *EULAR* European League Against Rheumatism, *SDAI* Simplified Disease Activity Index

**Table 3:** Treatment-emergent adverse events (safety population)

	CT-P17 (*N* = 324)	EU-adalimumab (*N* = 324)
Subjects with ≥ 1 TEAE, *n* (%)	169 (52.2)	184 (56.8)
Study drug-related	88 (27.2)	99 (30.6)
TEAEs reported in ≥ 5% of subjects in either treatment group		
ISR	16 (4.9)	22 (6.8)
Nasopharyngitis	17 (5.2)	20 (6.2)
Upper respiratory tract infection	17 (5.2)	20 (6.2)
Neutropenia	14 (4.3)	17 (5.2)
Subjects with ≥ 1 TESAE, *n* (%)	10 (3.1)	16 (4.9)
Subjects with ≥ 1 TEAE leading to study drug discontinuation, *n* (%)	5 (1.5)	8 (2.5)
Subjects with ≥ 1 TEAE classified as hypersensitivity/allergic reactions, *n* (%)	2 (0.6)	4 (1.2)
Subjects with ≥ 1 TEAE classified as ISR, *n* (%)	16 (4.9)	22 (6.8)
Subjects with ≥ 1 TEAE classified as infection, *n* (%)	97 (29.9)	103 (31.8)
Subjects with ≥ 1 TEAE classified as malignancy, *n* (%)	1 (0.3)^a^	0
Total number of TEAEs leading to death	0	0

Note: There were no significant differences between the CT-P17 and EU-adalimumab groups for any parameter (*p* > 0.05)

^a^Breast cancer that was considered unrelated to study drug; the subject’s family history of breast cancer was considered a risk factor by the investigator

*TEAE* treatment-emergent adverse event, *EU-adalimumab* European Union-approved adalimumab, *ISR* injection-site reaction, *TESAE* treatment-emergent serious adverse event

## Results

### Subject disposition and baseline characteristics

**Fig. 1:**
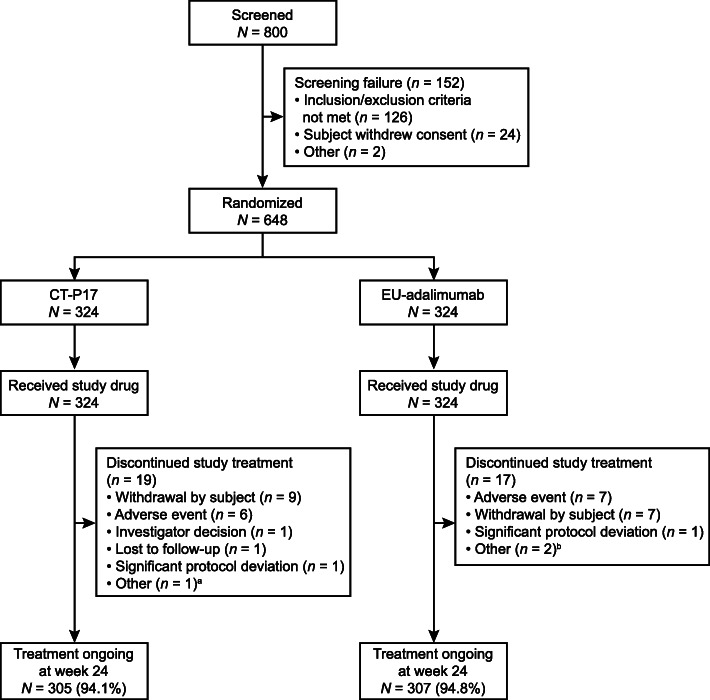
Subject disposition (ITT population). ^a^Subject discontinued CT-P17 treatment due to significant dose delay due to adverse event. ^b^Two subjects discontinued EU-adalimumab treatment due to subject decision due to adverse event. EU-adalimumab, European Union-approved adalimumab; ITT, intention-to-treat

Subjects were recruited between December 5, 2018, and April 25, 2019 (last subject week 24 visit: October 8, 2019). Of the 648 subjects who were randomized, 324 to each of the CT-P17 and EU-adalimumab groups (Fig. 1), 612 (94.4%) completed study treatment up to week 24 (CT-P17, 305/324 [94.1%]; EU-adalimumab, 307/324 [94.8%]). Nineteen (5.9%) and 17 (5.2%) subjects discontinued study treatment in the CT-P17 and EU-adalimumab groups, respectively. Withdrawal by subject was the most frequent reason for discontinuation (CT-P17, 9 [2.8%] subjects; EU-adalimumab, 7 [2.2%] subjects), followed by TEAEs (CT-P17, 6 [1.9%]; EU-adalimumab, 7 [2.2%]). Of the 36 (5.6%) subjects who discontinued study treatment, 25 (3.9%) terminated the study (CT-P17, 15 [4.6%]; EU-adalimumab, 10 [3.1%]) and 11 (1.7%) (CT-P17, 4 [1.2%]; EU-adalimumab, 7 [2.2%]) continued participation in the study.

Baseline demographics and disease characteristics were balanced between treatment groups (Table 1). Median age was 53.5 and 54.0 years for CT-P17 and EU-adalimumab, respectively. Most subjects were female (CT-P17, 76.9%; EU-adalimumab, 81.8%) and were mainly enrolled by sites in Eastern European countries, particularly Poland (231 [71.3%] subjects for both groups). Most subjects had high disease activity (SDAI score > 26). Stratification factors, SDAI score category, and country were well balanced between groups.

### Efficacy

#### Primary efficacy analysis

**Fig. 2:**
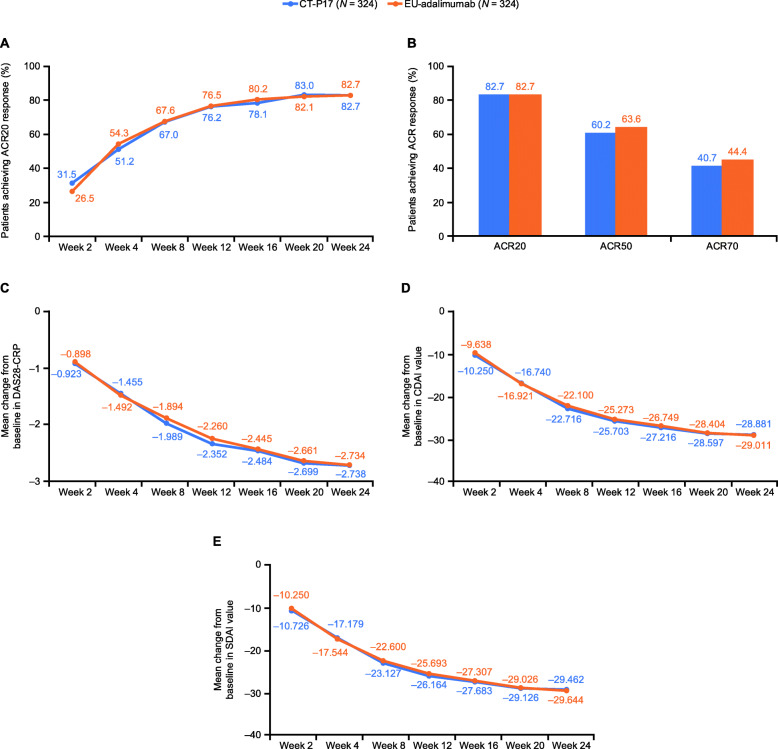
Secondary efficacy endpoints up to week 24 (ITT population). ACR20 response rate up to week 24 (**a**). ACR20, ACR50, and ACR70 response rates at week 24 (**b**). Mean change from baseline in DAS28-CRP up to week 24 (**c**). Mean change from baseline in CDAI value up to week 24 (**d**). Mean change from baseline in SDAI value up to week 24 (**e**). ACR, American College of Rheumatology; ACR20, 20% improvement according to American College of Rheumatology criteria; ACR50, 50% improvement according to American College of Rheumatology criteria; ACR70, 70% improvement according to American College of Rheumatology criteria; CDAI, Clinical Disease Activity Index; DAS28-CRP, Disease Activity Score in 28 joints C-reactive protein; EU-adalimumab, European Union-approved adalimumab; ITT, intention-to-treat; SDAI, Simplified Disease Activity Index

The ACR20 response rate at week 24 (Fig. 2a) was 82.7% (*n* = 268/324) for both the CT-P17 and EU-adalimumab groups (ITT population). The 95% confidence interval (CI) of − 5.94 to 5.94 for the estimate of treatment difference was entirely within the predefined equivalence margin of − 15 to 15% (EMA assumption), demonstrating therapeutic equivalence between treatment groups. Results for the PP population supported those for the ITT population (CT-P17, 87.0% [*n* = 248/285]; EU-adalimumab, 87.0% [*n* = 240/276]), with the 95% CI of − 5.60 to 5.78 for the estimate of treatment difference. Likewise, for both analysis populations, 90% CIs for the estimate of treatment difference (− 4.98 to 4.98 in the ITT population and − 4.70 to 4.86 in the PP population) were entirely within the asymmetric equivalence margin of − 12 to 15% (FDA assumption), thereby also demonstrating therapeutic equivalence between treatment groups. Sensitivity analysis using logistic regression with covariates for the primary endpoint provided similar results, − 5.75 to 5.86 (ITT population) and − 5.07 to 5.93 (PP population) for 95% CI.

#### Secondary and additional efficacy analyses

The proportions of subjects achieving ACR20, ACR50, and ACR70 responses up to week 24 were similar between the CT-P17 and EU-adalimumab groups (Fig. 2a, b; Supplementary Table 3, Additional file 1). Mean values of and change from baseline in DAS28-CRP (Fig. 2c), CDAI (Fig. 2d), and SDAI (Fig. 2e) were comparable between groups up to week 24, as were EULAR (CRP) response rates and SDAI, CDAI, DAS28 (CRP), and Boolean remission rates (Table 2). Mean hybrid ACR scores increased from week 2 to week 24 and were similar between groups (Supplementary Table 3, Additional file 1). Mean SF-36 physical and mental component scores increased from baseline to week 24; mean increases up to week 24 were similar between groups. At week 24, mean (SD) change from baseline in the SF-36 physical component score was 7.869 (7.4184) for CT-P17 (*n* = 309) and 8.213 (8.0179) for EU-adalimumab (*n* = 312). At week 24, mean (SD) change from baseline in the SF-36 mental component score was 5.879 (9.8480) for CT-P17 (*n* = 309) and 6.585 (9.7404) for EU-adalimumab (*n* = 312).

### Pharmacokinetic analysis

The mean adalimumab *C*_trough_ was comparable between groups in the PK population, although values were slightly higher in the CT-P17 than in the EU-adalimumab group (Supplementary Table 4, Additional file 1). Mean *C*_trough_ increased gradually from baseline to week 22 for both groups (Supplementary Figure 1, Additional file 1).

### Usability analysis

Mean scores for each domain of the PRE-SIAQ and POST-SIAQ were comparable between treatment groups (Supplementary Table 5, Additional file 1). All subjects in the population in which usability was assessed completed self-injection successfully.

### Safety

Overall, the proportion of subjects experiencing ≥ 1 TEAE was similar between treatment groups (CT-P17, 169 [52.2%] subjects; EU-adalimumab, 184 [56.8%] subjects) (Table 3). Most TEAEs were grade 1 or 2 in intensity. TEAEs considered by the investigator to be study drug-related were reported by 187 (28.9%) subjects, with similar proportions between treatment groups (CT-P17, 88 [27.2%]; EU-adalimumab, 99 [30.6%]) (Table 3). A similar proportion of subjects in each group experienced TEAEs leading to study drug discontinuation; of these, 2 (0.6%) and 4 (1.2%) subjects, respectively, experienced TEAEs that were considered study drug related. The most frequently reported TEAEs in the CT-P17 group were nasopharyngitis and upper respiratory tract infection (17 [5.2%] subjects each) and ISR (16 [4.9%]); in the EU-adalimumab group, these were ISR (22 [6.8%]) and nasopharyngitis and upper respiratory tract infection (20 [6.2%] each) (Table 3). Treatment-emergent serious adverse events (TESAEs) were reported for 10 (3.1%) subjects in the CT-P17 group and 16 (4.9%) in the EU-adalimumab group (Table 3). Study drug-related TESAEs were reported for 9 subjects overall (CT-P17, 5 [1.5%]; EU-adalimumab, 4 [1.2%]) (Supplementary Table 6, Additional file 1). No deaths were reported up to week 24.

TEAEs classified as hypersensitivity/allergic reactions, ISRs, or infections were experienced by similar proportions of subjects in each group (Table 3). Conversion to positive interferon-γ release assay, up to week 24, was experienced by 12 (3.7%) and 17 (5.2%) subjects in the CT-P17 and EU-adalimumab groups, respectively. Latent tuberculosis was reported in 12 (3.7%) and 15 (4.6%) subjects in the CT-P17 and EU-adalimumab groups, respectively; for 7 (2.2%) and 10 (3.1%) of these subjects, this was considered study drug-related. All these subjects began tuberculosis prophylaxis, except for the single subject in each group who terminated study participation. Two (0.6%) subjects in the EU-adalimumab group reported active tuberculosis and discontinued study treatment. One (0.3%) subject experienced a TEAE classified as malignancy (breast cancer) in the CT-P17 group (Table 3); however, the site investigator considered this event to be unrelated to study drug.

Small imbalances were identified in the proportions of subjects reporting TEAEs classified in the SOC of gastrointestinal disorders (CT-P17, 24 [7.4%] subjects; EU-adalimumab, 16 [4.9%] subjects), nervous system disorders (CT-P17, 19 [5.9%] subjects; EU-adalimumab, 9 [2.8%] subjects), and metabolism and nutrition disorders (CT-P17, 12 [3.7%] subjects; EU-adalimumab, 6 [1.9%] subjects) (Supplementary Table 7, Additional file 1). However, among these SOCs, other than for two grade 3 TEAEs that were considered unrelated to study drug in the EU-adalimumab group (1 nervous system disorder of carotid arteriosclerosis, and 1 metabolism and nutrition disorder of hypertriglyceridemia), all TEAEs were grade 1–2 in intensity.

Mean (SD) 100-mm VAS scores for local site pain measurements decreased over time. Results at each visit were comparable between treatment groups. At week 0, mean (SD) VAS scores for local site pain were 6.64 (10.887) for CT-P17 and 4.85 (7.300) for EU-adalimumab, while mean VAS scores at week 24 had decreased to 4.45 (8.393) for CT-P17 and to 4.32 (8.651) for EU-adalimumab.

### Immunogenicity

At baseline, 11 (3.4%) and 6 (1.9%) subjects were ADA-positive and 4 (1.2%) and 1 (0.3%) subjects were NAb-positive in the CT-P17 and EU-adalimumab groups, respectively (Supplementary Table 8, Additional file 1). At week 24, 93 (28.7%) and 116 (35.8%) subjects were ADA-positive and 83 (25.6%) and 103 (31.8%) subjects were NAb-positive in the CT-P17 and EU-adalimumab groups, respectively. Overall, immunogenicity was slightly lower numerically for the CT-P17 group than for the EU-adalimumab group throughout the study.

### ADA subgroup analysis

The proportions of subjects achieving an ACR20 response at week 24 were similar between the CT-P17 and the EU-adalimumab treatment groups in both the ADA-positive and ADA-negative subgroups (Supplementary Table 9, Additional file 1). Mean *C*_trough_ was lower in the ADA-positive subgroup than in the ADA-negative subgroup for both treatment groups (Supplementary Figure 1 and Supplementary Table 4, Additional file 1). A lower proportion of subjects in the ADA-negative subgroup experienced ≥1 TEAE than did those in the ADA-positive subgroup (49.7% and 59.8%, respectively); however, with a limited number of events, there was no apparent correlation between the presence of ADA and the incidence of TESAEs, TEAEs classified as hypersensitivity/reactions, or TEAEs classified as ISRs (Supplementary Table 10, Additional file 1).

## Discussion

This study demonstrated equivalent efficacy of CT-P17 to EU-adalimumab in the proportion of subjects achieving an ACR20 response at week 24. This primary endpoint was met in both the ITT and PP analysis populations for both the EMA (− 15 to 15%, 95% CI) and the FDA (− 12 to 15%, 90% CI) statistical assumptions. Comparable efficacy of CT-P17 and EU-adalimumab was demonstrated for secondary endpoints up to week 24. PK parameters were also comparable between groups, although mean *C*_trough_ was slightly higher for CT-P17 than for EU-adalimumab. The usability of CT-P17, assessed by ability to complete successful self-injections and subject-reported outcomes, was comparable to that of EU-adalimumab. The overall safety profile of CT-P17 was similar to that of EU-adalimumab, although there were small imbalances between the CT-P17 and EU-adalimumab groups for some SOCs.

In our study, the ACR20 response rate at week 24, using high-concentration (100 mg/ml) formulations of CT-P17 and EU-adalimumab, was 82.7% in both treatment groups (ITT population). These observed ACR20 response rates are only slightly above the range of ACR20 responses for reference adalimumab (50 mg/ml) and adalimumab biosimilars in other adalimumab biosimilar studies, in which ACR20 response rates at week 24 or week 26 ranged between 63.9 and 82.5% (Supplementary Figure 2, Additional file 1) [21–25]. However, our study did not compare the high-concentration formulation to the low-concentration formulation of adalimumab.

We also investigated the influence of immunogenicity status on the proportion of subjects achieving an ACR20 response at week 24. Overall, a slightly lower proportion of ADA-positive subjects achieved an ACR20 response at week 24 than did ADA-negative subjects (81.4% vs 85.1%), consistent with previous reports for reference adalimumab and adalimumab biosimilars [13, 26]. However, within subject subgroups by ADA status, ACR20 response rates were similar between CT-P17 and EU-adalimumab treatment groups, in line with similar data reported previously for adalimumab biosimilars [26, 27].

In this study, we observed slightly higher mean *C*_trough_ values in the CT-P17 group compared with the EU-adalimumab group. In a separate study, ADA formation was associated with increased clearance and lower serum adalimumab concentrations [13]. However, in this study, AUC and clearance were not evaluated, so this could not be examined directly. It is possible that the differences may be associated with the lower proportion of ADA-positive subjects in the CT-P17 versus EU-adalimumab group. Indeed, in our study, *C*_trough_ was generally lower in the ADA-positive than in the ADA-negative subgroup; *C*_trough_ values became more similar between groups when compared within these subgroups. Nevertheless, *C*_trough_ values of the ADA-positive subgroup were within the therapeutic level of adalimumab (5 to 8 μg/ml) [13]*.* In addition, our findings are in keeping with previous reports for adalimumab biosimilars [26, 28].

The overall safety profile of CT-P17 in this study was consistent with the known safety profile of reference adalimumab [13]. In both treatment groups, the most frequently reported study drug-related TEAEs were ISRs, consistent with information provided in the EU-adalimumab summary of product characteristics [13]. ISRs, as well as the other protocol-specified TEAESIs of hypersensitivity/allergic reactions and infections, were experienced by similar proportions of subjects in each treatment group. There were small imbalances, at the SOC level, in the proportions of subjects reporting TEAEs for gastrointestinal disorders, nervous system disorders, and metabolism and nutrition disorders. These TEAEs were mostly grade 1–2 in intensity and, at the Preferred Term level, absolute differences between groups in the number of subjects experiencing a given TEAE were small. When analyzed by ADA status, the incidence of TEAEs, but not of TESAEs, was higher for ADA-positive than for ADA-negative subjects; there was no apparent correlation between the presence of ADAs and hypersensitivity/allergic reactions or ISRs.

This is the first report of a phase III clinical trial comparing CT-P17 to reference adalimumab. Strengths of this study include its randomized, double-blind design, and the use of well-established outcome measures. Equivalent efficacy of CT-P17 and EU-adalimumab was established using both symmetric and asymmetric equivalence margins, as agreed to by the EMA and the FDA, respectively. In addition to the data reported from comparing these treatments up to week 24, this study will provide valuable information regarding the efficacy and safety of transitioning from EU-adalimumab to CT-P17 (during treatment period 2).

Limitations of this study include the relatively short follow-up period (24 weeks) reported herein; however, this study is ongoing and efficacy and safety data up to week 52 will be reported. Although sufficient testing was done for regulatory purposes, comprehensive PK data were not collected. While treatment groups were well balanced by stratification factors including country, subjects from Eastern European countries, particularly Poland, accounted for most of the study population. The races of subjects enrolled in this study included white, mestizo, and Native Peruvian. This could limit the global generalizability of the findings; however, this should be viewed in the context of the global scope of the CT-P17 clinical development program that included American Indian or Alaska Native, Black or African American, and Asian subjects, among which there were no differences in clinical responses [11, 16, 29]. Also, since this was a comparative study to demonstrate equivalence of CT-P17 to EU-adalimumab, it did not aim to evaluate the drugs across other ethnic groups.

The original reference adalimumab was developed as a low-concentration (50 mg/ml) formulation containing citrate. Subsequently, a high-concentration (100 mg/ml), citrate-free formulation of reference adalimumab has been developed [13, 14]. While the high-concentration CT-P17 formulation is similar to the newer formulation of reference adalimumab [13, 14], it differs from the low-concentration (50 mg/ml) adalimumab biosimilars that are currently approved [12, 30–33]. The high-concentration formulations of both CT-P17 and reference adalimumab offer the potential to administer high-dose (80 mg/0.8 ml) induction treatment to patients with inflammatory bowel disease with a reduced number of injections. In addition, the citrate-free buffer may benefit patients by reducing discomfort during injection [12, 15].

## Conclusions

In conclusion, demonstration of equivalent efficacy and comparable safety and immunogenicity of CT-P17 to EU-adalimumab in this study support the ongoing clinical evaluation of CT-P17 as an adalimumab biosimilar.

## Supplementary Information

**Additional file 1: **Supplementary methods, including **Table S1** Study centers and IRB/IEC information, Full inclusion and exclusion criteria, **Table S2** Schedule of assessments (screening and treatment period 1), and Analysis populations. **Table S3** ACR50 and ACR70 response rates and hybrid ACR scores up to week 24 (ITT population). **Table S4** Mean (SD) C_trough_ overall and by ADA status (PK population). **Figure S1** Mean (±SD) C_trough_ by ADA status (PK population). **Table S5** Mean (SD) scores by domain for PRE-SIAQ and POST-SIAQ (usability population). **Table S6** Study drug–related TESAEs (safety population). **Table S7** TEAEs by System Organ Class reported by ≥2% of subjects in either treatment group (safety population). **Table S8** Summary of immunogenicity results (safety population). **Table S9** ACR20 response rate at week 24 by ADA status (ITT population). **Table S10** Treatment-emergent adverse events by ADA status (safety population). **Figure S2** Historical data for ACR20 response rate at week 24 for reference or biosimilar adalimumab (50 mg/ml), compared with CT-P17 or reference adalimumab (100 mg/ml) treatment in the current study (ITT population).

## Data Availability

Available data and methodological information for this ongoing study are included in this article and accompanying supplementary materials.

## Abbreviations

ACR: American College of Rheumatology
ACR20: 20% improvement according to American College of Rheumatology criteria
ADA: Anti-drug antibody
bDMARD: Biological disease-modifying antirheumatic drug
bsDMARD: Biosimilar disease-modifying antirheumatic drug
CDAI: Clinical Disease Activity Index
CI: Confidence interval
CRO: Contract Research Organization
CRP: C-reactive protein
csDMARD: Conventional synthetic disease-modifying antirheumatic drug
*C*_trough_: Trough serum adalimumab concentration
DAS28: Disease Activity Score in 28 joints
EMA: European Medicines Agency
EU-adalimumab: European Union-approved adalimumab
EULAR: European League Against Rheumatism
ESR: Erythrocyte sedimentation rate
FDA: US Food and Drug Administration
ISR: Injection-site reaction
ITT: Intention-to-treat
IWRS: Interactive web response system
NAb: Neutralizing antibody
PK: Pharmacokinetics
PP: Per-protocol
q2w: Every 2 weeks
SDAI: Simplified Disease Activity Index
SF-36: 36-item Short Form Health Survey
SIAQ: Self-Injection Assessment Questionnaire
SOC: System Organ Class
TEAE: Treatment-emergent adverse event
TEAESI: Treatment-emergent adverse event of special interest
TESAE: Treatment-emergent serious adverse event
TNF: Tumor necrosis factor
VAS: Visual analog scale
