# Supplementary material for: Efficacy and safety of biosimilar CT-P17 versus reference adalimumab in subjects with rheumatoid arthritis: 24-week results from a randomized study
Source: Arthritis Res Ther. 2021 Feb 5;23:51. doi: 10.1186/s13075-020-02394-7 (PMC7863328; doi:10.1186/s13075-020-02394-7)
Supplement: Supplementary file 1 — Additional file 1: Supplementary methods, including Table S1 Study centers and IRB/IEC information, Full inclusion and exclusion criteria, Table S2 Schedule of assessments (screening and treatment period 1), and Analysis populations. Table S3 ACR50 and ACR70 response rates and hybrid ACR scores up to week 24 (ITT population). Table S4 Mean (SD) Ctrough overall and by ADA status (PK population). Figure S1 Mean (±SD) Ctrough by ADA status (PK population). Table S5 Mean (SD) scores by domain for PRE-SIAQ and POST-SIAQ (usability population). Table S6 Study drug–related TESAEs (safety population). Table S7 TEAEs by System Organ Class reported by ≥2% of subjects in either treatment group (safety population). Table S8 Summary of immunogenicity results (safety population). Table S9 ACR20 response rate at week 24 by ADA status (ITT population). Table S10 Treatment-emergent adverse events by ADA status (safety population). Figure S2 Historical data for ACR20 response rate at week 24 for reference or biosimilar adalimumab (50 mg/ml), compared with CT-P17 or reference adalimumab (100 mg/ml) treatment in the current study (ITT population). [file 13075_2020_2394_MOESM1_ESM.docx]

Additional file 1

Efficacy and Safety of Biosimilar CT-P17 versus Reference Adalimumab in Subjects with Rheumatoid Arthritis: 24-Week Results from a Randomized Study^[[1]](#footnote-1)^

Jonathan Kay, MD,^1^ Janusz Jaworski, MD,^2^ Rafal Wojciechowski, MD,^3^ Piotr Wiland, MD,^4^ Anna Dudek, MD,^5^ Marek Krogulec, MD,^6^ Slawomir Jeka, MD,^7^ Agnieszka Zielinska, MD,^8^ Jakub Trefler, MD, PhD,^9^ Katarzyna Bartnicka-Maslowska, MD,^10^ Magdalena Krajewska-Wlodarczyk, MD,^11^ Piotr A. Klimiuk, MD,^12^ Sang Joon Lee, PhD,^13^ Yun Ju Bae, MS,^13^ Go Eun Yang, BS,^13^ Jae Kyoung Yoo, BS,^13^ Daniel E. Furst, MD,^14–16*^ and Edward Keystone, MD^17*,^†

^1^University of Massachusetts Medical School and UMass Memorial Medical, Worcester, MA, USA; ^2^Reumatika-Centrum Reumatologii, Warsaw, Poland; ^3^University Hospital No 2, Bydgoszcz, Poland; ^4^Medical University, Wroclaw Poland; ^5^Centrum Medyczne AMED, Warsaw, Poland; ^6^Rheumatology Clinic NZOZ Lecznica MAK-MED, Nadarzyn, Poland; ^7^Nasz Lekarz Przychodnie Medyczne, Toruń, Poland; ^8^Medycyna Kliniczna Marzena Waszczak-Jeka, Warsaw, Poland; ^9^Reuma Centrum, Warsaw, Poland; ^10^Centrum Medyczne AMED Oddzial w Lodzi, Łódź, Poland; ^11^University of Warmia and Mazury, Olsztyn, Poland; ^12^Medical University of Białystok and Gabinet Internistyczno-Reumatologiczny Piotr Adrian Klimiuk, Białystok, Poland; ^13^Celltrion Inc, Incheon, Republic of Korea; ^14^University of California, Los Angeles, CA, USA; ^15^University of Washington, Seattle, WA, USA; ^16^University of Florence, Florence, Italy; ^17^Mount Sinai Hospital, University of Toronto, Toronto, Canada

**CONTENTS**

[SUPPLEMENTARY METHODS 2](#_Toc45621804)

[Study centers 2](#_Toc45621805)

[**Supplementary Table 1.** Study centers and IRB/IEC information 2](#_Toc45621806)

[Full inclusion and exclusion criteria 5](#_Toc45621807)

[**Supplementary Table 2.** Schedule of assessments (screening and treatment period 1) 11](#_Toc45621808)

[Analysis populations 13](#_Toc45621809)

[SUPPLEMENTARY RESULTS 14](#_Toc45621810)

[**Supplementary Table 3.** ACR50 and ACR70 response rates and hybrid ACR scores up to week 24 (ITT population) 14](#_Toc45621811)

[**Supplementary Table 4.** Mean (SD) C_trough_ overall and by ADA status (PK population) 15](#_Toc45621812)

[**Supplementary Figure 1.** Mean (±SD) C_trough_ by ADA status (PK population). 16](#_Toc45621818)

[**Supplementary Table 5.** Mean (SD) scores by domain for PRE-SIAQ and POST-SIAQ (usability population) 17](#_Toc45621813)

[**Supplementary Table 6.** Study drug–related TESAEs (safety population) 19](#_Toc45621814)

[**Supplementary Table 7.** TEAEs by System Organ Class reported by ≥2% of subjects in either treatment group (safety population) 20](#_Toc45621815)

[**Supplementary Table 8.** Summary of immunogenicity results (safety population) 21](#_Toc45621816)

[**Supplementary Table 9.** ACR20 response rate at week 24 by ADA status (ITT population) 22](#_Toc45621817)

[**Supplementary Table 10.** Treatment-emergent adverse events by ADA status (safety population) 23](#_Toc45621819)

[SUPPLEMENTARY MATERIALS FOR DISCUSSION 24](#_Toc45621820)

[**Supplementary Figure 2.** Historical data for ACR20 response rate at week 24 for reference or biosimilar adalimumab (50 mg/ml), compared with CT-P17 or reference adalimumab (100 mg/ml) treatment in the current study (ITT population) 24](#_Toc45621821)

[REFERENCES FOR SUPPLEMENTARY MATERIAL 25](#_Toc45621822)

# SUPPLEMENTARY METHODS

## Study centers

**Supplementary Table 1.** Study centers and IRB/IEC information

| Country | Study center | IRB/IEC |
| --- | --- | --- |
| Bulgaria | National Multiprofile Transport Hospital Tsar Boris III, Sofia | Ethics Committee for Multicentre Trials  Sofia |
|  | University Multiprofile Hospital for Active Treatment Kaspela, Plovdiv |  |
|  | Multiprofile Hospital for Active Treatment Plovdiv, Plovdiv |  |
|  | Diagnostic-Consultative Center Aleksandrovska EOOD, Sofia |  |
| Hungary | MÁV Kórház és Rendelőintézet Szolnok, Szolnok | Egészségügyi Tudományos Tanács Klinikai Farmakológiai Etikai Bizottsága, Budapest and Országos Gyógyszerészeti és Élelmezés-egészségügyi Intézet (OGYÉI), Budapest |
|  | Csongrád Megyei Dr. Bugyi István Kórház, Szentes |  |
|  | Pest Megyei Flór Ferenc Kórház, Kistarcsa |  |
|  | QUALICLINIC Kft, Budapest |  |
|  | Vital Medical Center, Veszprem |  |
|  | DRC Gyogyszervizsgalo Kozpontot, Balatonfured |  |
| Lithuania | PI Republican Siauliai Hospital, Siauliai | Lithuanian Bioethics Committee, Vilnius |
|  | Klaipedos Universitetine Ligonine, Klaipeda |  |
|  | Hospital of Lithuanian University of Health Sciences Kaunas Clinics, Kaunas |  |
| Peru | Hospital Maria Auxiliadora, Lima | Comité Institucional de Bioética (CIB) de Vía Libre, Lima |
|  | Clínica Internacional - PPDS, Lima |  |
|  | Hospital Militar Central, Jesús Maria |  |
|  | Instituto de Ginecologia Y Reproduccion - PPDS, Santiago De Surco, Lima |  |
|  | Hogar Clínica San Juan de Dios, Avenida Ejercito Cayma, Arequipa |  |
|  | Bio Ciencias Peru Sociedad Comercial de Responsabilidad Limitada, Pueblo Libre |  |
|  | Clínica Médica Cayetano Heredia, Lima |  |
|  | Instituto Peruano del Hueso y la Articulacion SAC, San Isidro, Lima |  |
|  | Instituto del Cerebro y la Columna Vertebral SAC, Miraflores, Lima |  |
|  | Hospital Nacional Edgardo Rebagliati Martins, Lima | Comité Institucional de Ética en Investigación del Hospital Nacional Alberto Sabogal Sologuren, Bellavista, Callao |
| Poland | REUMA CENTRUM, REUMATOLOG WARSZAWA Specjalistyczna Praktyka lekarska, Warszawa | Komisja Bioetyczna przy Kujawsko-Pomorskiej Okregowej Izbie Lekarskiej w Toruniu, Toruń, Kujawsko-Pomorskie |
|  | Prywatna Praktyka Lekarska Pawel Hrycaj, Poznan |  |
|  | Gabinet Internistyczno-Reumatologiczny Piotr Adrian Klimiuk, Białystok |  |
|  | REUMATIKA - Centrum Reumatologii NZOZ, Warszawa |  |
|  | Szpital Uniwersytecki Nr 2 im. Dr Jana Biziela w Bydgoszczy, Bydgoszcz |  |
|  | Zespol Poradni Specjalistycznych REUMED, Lublin |  |
|  | Centrum Medyczne AMED, Warszawa |  |
|  | NZOZ Lecznica MAK-MED, Nadarzyn |  |
|  | Nasz Lekarz Przychodnie Medyczne, Toruń |  |
|  | MCM Krakow - PRATIA - PPDS, Krakow |  |
|  | Centrum Medyczne AMED Oddzial w Lodzi, Łódź |  |
|  | Samodzielny Publiczny Zespol Opieki Zdrowotnej w Tomaszowie Lubelskim, Tomaszow Lubelski |  |
|  | Niepubliczny Zaklad Opieki Zdrowotnej Biogenes Sp. z o.o., Wroclaw |  |
|  | Medycyna Kliniczna Marzena Waszczak-Jeka, Warszawa |  |
|  | ETYKA Osrodek Badan Klinicznych, Olsztyn |  |
|  | Korczowski Bartosz, Gabinet Lekarski, Rzeszow |  |
|  | K2J2 Medical Center, Wolomin |  |
| Ukraine | Medical Center of LLC Medical Center Konsylium Medikal, Kyiv | Commission on Ethics Questions of LLC Medical Center Konsylium Medikal, Kyiv |
|  | SI National Scientific Center Institute of Cardiology n.a acad. M.D.Strazhesko of NAMS of Ukraine, Kyiv | Commission on Ethics Questions of National Scientific Center Institute of Cardiology n.a MD Strazhesko of NAMS of Ukraine, Kyiv |
|  | Municipal Non-profit Enterprise “Ternopil University Hospital” of Ternopil Regional Council, Ternopil | Commission on Ethics Questions of SRI of Invalid Rehab. (ESTC) of VNMU n.a. M.I. Pyrohov, Ternopil |
|  | Clinic of SRI of Invalid Rehab. (ESTC) of VNMU n.a. M.I.Pyrohov, Vinnytsia | Commission on Ethics Questions of SRI of Invalid Rehab. (ESTC) of VNMU n.a. M.I. Pyrohov, Vinnytsia |
|  | ME Poltava Reg.Clin.Hospital n.a.M.V.Skliphosovskyi of Poltava Reg.Council, Poltava | Commission on Ethics Questions of ME Poltava Regional Clinical Hospital n.a. M.V. Skliphosovskyi of PRC, Poltava |
|  | Municipal Institution of Kyiv Regional Council Kyiv Regional Clinical Hospital, Kiev | Commission on Ethics Questions of City Clinical Hospital #1, Kyiv |
|  | Communal Non-Commercial Enterprise "Vinnytsia City Clinical Hospital №1," Vinnytsia | Commission on Ethics Questions of City Clinical Hospital #1, Vinnytsia |
|  | Vinnytsia Regional Clinical Hospital named after N.I. Pirogov Vinnytsia Regional Council, Vinnytsia | Commission on Ethics Questions of Vinnytsia Regional Clinical Hospital n.a. M. I. Pyrohov, Vinnytsia |
|  | Municipal Institution of Sumy Regional Council Sumy Regional Clinical Hospital, Sumy | Commission on Ethics Questions of Sumy Regional Clinical Hospital, Sumy |
|  | Communal Nonprofit Enterprise Regional Clinical Hospital of the Ivano-Frankivsk Regional Council, Ivano-Frankivsk | Commission on Ethics Questions of Regional Clinical Hospital, Ivano-Frankivsk |
|  | Kyiv City Clinical Hospital #3, Kyiv | Commission on Ethics Questions of Kyiv City Clinical Hospital #3, Kyiv |
|  | Municipal Nonprofit Enterprise of Kharkiv Regional Council Regional Clinical Hospital, Kharkiv | Commission on Ethics Questions of Municipal Non-Profit Enterprise of Kharkiv Regional Council "Regional Clinical Hospital," Kharkiv |

*IEC* Independent Ethics Committee, *IRB* Institutional Review Board

## Full inclusion and exclusion criteria

Each subject had to meet all of the following criteria to be enrolled in this study:

1. Subject was male or female 18–75 years old (inclusive).
2. Subject had a diagnosis of rheumatoid arthritis (RA) according to the 2010 American College of Rheumatology (ACR)/European League Against Rheumatism (EULAR) classification criteria [1] for ≥24 weeks prior to the first administration of study drug (day 1).
3. Subject had active disease as defined by the presence of ≥6 swollen joints (of 66 assessed), ≥6 tender joints (of 68 assessed), and either an erythrocyte sedimentation rate (ESR) >28 mm/hour or a serum C-reactive protein (CRP) concentration >1.0 mg/dl (>10 mg/l) at screening.
4. Subject had been receiving oral or parenteral methotrexate (MTX) at a dose of 12.5–25 mg/week, or 10 mg/week if intolerant to a higher dose, for ≥12 weeks and had been on a stable dose and route of MTX for ≥4 weeks prior to day 1.
5. Subject had adequate hepatic and renal function at screening as defined by the following clinical chemistry results:
   - Serum creatinine ≤1.5 × upper limit of normal (ULN) or an estimate creatinine clearance level >50 ml/min (by Cockcroft–Gault formula) (Système International d’Unites [SI] units: 0.84 ml/s)
   - Serum alanine aminotransferase ≤3.0 × ULN
   - Serum aspartate aminotransferase ≤3.0 × ULN
   - Serum total bilirubin ≤1.5 × ULN
6. Subject had the following hematology laboratory test results at screening:
   - Hemoglobin >8.0 g/dl (SI units: >80 g/l or 4.96 mmol/l)
   - Absolute neutrophil count ≥1.5 × 10^3^ cells/μl (SI units: ≥1.5 × 10^9^ cells/l)
   - Platelet count ≥75 × 10^3^ cells/μl (SI units: ≥75 × 10^9^ cells/l)
7. Subject (or legal guardian, if applicable) had been informed of the full nature and purpose of the study, including possible risks and side effects, had the ability to cooperate with the investigator and been given ample time and opportunity to read and understand verbal and/or written instructions, and signed the written informed consent form (ICF) with date prior to participation in the study.
8. Subject and their partner of childbearing potential had to agree to use a highly effective method of contraception throughout the study and for 6 months after the last dose of assigned treatment. Examples include the following:
   - Hormonal contraceptives (combined or progestogen-only) associated with inhibition of ovulation
   - Intrauterine devices
   - Sexual abstinence (not periodically, but for the entire period of risk).

A man or woman was of childbearing potential if, in the opinion of the investigator, he or she was biologically capable of having children and was sexually active. Male and female subjects and their partners who had been surgically sterilized for <24 weeks prior to the date of informed consent had to agree to use any medically acceptable methods of contraception. Menopausal females had to experience their last period >1 year prior to the date of informed consent to be classified as not of childbearing potential.

1. Subject had to be able and willing to self-administer subcutaneous (SC) injections or designate a qualified person(s) to administer SC injection.

Any subject meeting any of the following criteria was excluded from the study:

1. Subject had previously received investigational or licensed product; biologic or targeted synthetic disease-modifying antirheumatic drugs (DMARDs; e.g. tofacitinib, baricitinib) for the treatment of RA and/or tumor necrosis factor (TNF) inhibitors for any purpose.
2. Subject had allergies to any of the excipients of study drug or any other murine and human proteins, or subject with a hypersensitivity to immunoglobulin products.
3. Subject currently had or had a history of any of the following infections:
   - A known infection with hepatitis B (active or carrier of hepatitis B), hepatitis C, or infection with human immunodeficiency virus (HIV). However, a subject with past hepatitis B infection was allowed if resolved.
   - Acute infection requiring oral antibiotics ≤2 weeks or parenteral injection of antibiotics ≤4 weeks prior to day 1.
   - Recurrent herpes zoster or other chronic or recurrent infection ≤6 weeks prior to day 1.
   - Past or current granulomatous infections or other severe or chronic infections (e.g. sepsis, abscess, opportunistic infections, or invasive fungal infections such as histoplasmosis). A subject who had a past diagnosis with sufficient documentation of complete resolution of the infection could be enrolled in the study.
   - Other serious infections ≤24 weeks prior to day 1.
4. Subject currently had or had a history of any of the following tuberculosis conditions:
   - Subject had a history of tuberculosis or a current diagnosis of tuberculosis. A subject who had a previous diagnosis of active tuberculosis could not be enrolled in the study even if there was sufficient documentation of complete resolution of active tuberculosis.
   - Subject had exposure to a person with active tuberculosis, such as first-degree family members or co-workers.
   - Subject had an indeterminate result for interferon-γ release assay (IGRA) or latent tuberculosis (defined as a positive result of IGRA with a negative examination of chest X-ray) at screening. A subject who had a previous diagnosis of latent tuberculosis could not be enrolled despite sufficient documentation of prophylaxis. If the result of the IGRA was indeterminate at screening, 1 retest would be possible during the screening period. If the repeated IGRA result was indeterminate again or positive, the subject would be excluded from the study. If the repeated IGRA test result was negative, the subject could be enrolled in the study.
5. Subject had a medical condition including one or more of the following:
   - Classified as Class II or III obese by World Health Organization classification (body mass index ≥35 kg/m^2^).
   - Uncontrolled diabetes mellitus, even after insulin treatment.
   - Uncontrolled hypertension (as defined by systolic blood pressure [BP] ≥160 mmHg or diastolic BP ≥100 mmHg).
   - Any other inflammatory or rheumatic diseases, including but not limited to psoriatic arthritis, ankylosing spondylitis, spondyloarthritis, systemic lupus erythematosus, Lyme disease, or fibromyalgia, that may confound the evaluation of the effect of the study drug.
   - Significant systemic RA involvement (e.g. Sjögren’s syndrome, vasculitis, pulmonary fibrosis) that would put the subject at risk if they were enrolled.
   - A known malignancy ≤5 years prior to day 1 except completely excised and cured squamous carcinoma of the uterine cervix in situ, cutaneous basal cell carcinoma, or cutaneous squamous cell carcinoma.
   - New York Heart Association Class III or IV heart failure, severe uncontrolled cardiac disease (unstable angina or clinically significant electrocardiogram abnormalities), or myocardial infarction ≥24 weeks prior to day 1.
   - History of organ transplantation, including corneal graft/transplantation.
   - Any clinically significant respiratory disease, including but not limited to chronic obstructive pulmonary disease, asthma, or pleural effusion.
   - Previous diagnosis of symptoms suggestive of demyelinating disorders, including multiple sclerosis, and Guillain-Barré syndrome.
   - Any conditions significantly affecting the nervous system (e.g. neuropathic conditions or nervous system damage) if it could interfere with the investigator’s assessment on disease activity scores including joint counts.
   - Any other serious acute or chronic medical or psychiatric condition that could increase the risk associated with study participation or study drug administration or that could interfere with the interpretation of study results.
   - History or evidence of any other clinically significant disorder, condition, or disease that, in the opinion of the investigator, would pose a risk to subject safety or interfere with the study evaluation, procedures, or completion.
6. Subject had received or planned to receive any of the following prohibited medications or treatment:
   - Intra-articular corticosteroids ≤4 weeks prior to day 1. Subjects were permitted to receive either oral or parenteral glucocorticoids (≤10 mg daily prednisone/prednisolone or equivalent), and nonsteroidal anti-inflammatory drug, if they had received a stable dose for ≥4 weeks prior to day 1 and the same dose was maintained until the primary endpoint assessment at week 24. In addition, subjects were permitted to receive low-potency topical, otic, and ophthalmic glucocorticoid preparations provided the preparations were administered per the instructions on the product label.
   - Conventional DMARDs, other than MTX, including hydroxychloroquine, chloroquine, or sulfasalazine, ≤4 weeks prior to day 1. Subjects who had discontinued leflunomide and had successful chelation with 8 g cholestyramine (3 times daily) for 11 days had to wait 4 weeks after the last dose of cholestyramine prior to day 1. Subjects who discontinued leflunomide and did not have a cholestyramine washout had to wait 12 weeks after the last dose of leflunomide prior to day 1.
   - Any other investigational device or medical product ≤4 weeks prior to day 1 or 5 half-lives, whichever was longer.
   - Alkylating agents ≤1 year prior to day 1.
   - Herbal products ≤2 weeks prior to day 1.
   - Live or live-attenuated vaccine ≤4 weeks prior to day 1, or any planned live or live-attenuated vaccination during the study period.
   - Any surgical procedure, including bone or joint surgery or synovectomy (including joint fusion or replacement) ≤12 weeks prior to day 1 or planned ≤24 weeks after day 1.
7. Subject had severe physical incapacitation (severely limited in ability to perform routine self-care, had RA ACR global functional status Class IV [2], or who could not benefit from medication).
8. Female subjects who were pregnant or breastfeeding or planned to become pregnant or breastfeed within 6 months of the last dose of study drug.
9. Subject had current or past history of drug or alcohol abuse ≤2 years from screening.
10. Subject who, in the opinion of their general practitioner or investigator, should not have participated in the study.

**Supplementary Table 2.** Schedule of assessments (screening and treatment period 1)

|  | Screening | Treatment period 1 | | | | | | | | |
| --- | --- | --- | --- | --- | --- | --- | --- | --- | --- | --- |
|  |  | Dose 1 | Dose 2 | Dose 3 | Dose 4 | Dose 5 | Dose 7 | Dose 9 | Dose 11 | Dose 13 |
| Study visit (week)* | −6 | 0 | 2 | 4 | 6 | 8 | 12 | 16 | 20 | 24 |
| Study visit (day)* | −42 to −1 | 1 | 15 | 29 | 43 | 57 | 85 | 113 | 141 | 169 |
| Informed consent | X |  |  |  |  |  |  |  |  |  |
| Demographics, height, medical history | X |  |  |  |  |  |  |  |  |  |
| Hepatitis B, hepatitis C, and HIV test | X |  |  |  |  |  |  |  |  |  |
| Serum pregnancy test | X |  |  |  |  |  |  |  |  |  |
| Chest X-ray | X |  |  |  |  |  |  |  |  |  |
| IGRA | X |  |  |  |  |  | X |  |  | X |
| Inclusion/exclusion criteria | X | X |  |  |  |  |  |  |  |  |
| Randomization |  | X |  |  |  |  |  |  |  |  |
| Efficacy assessments—predose |  |  |  |  |  |  |  |  |  |  |
| Swollen joint count (66 joints/28 joints) | X | X | X | X |  | X | X | X | X | X |
| Tender joint count (66 joints/28 joints) | X | X | X | X |  | X | X | X | X | X |
| VAS pain score | X | X | X | X |  | X | X | X | X | X |
| VAS global assessment of disease activity (subject/physician) scores | X | X | X | X |  | X | X | X | X | X |
| Health Assessment Questionnaire | X | X | X | X |  | X | X | X | X | X |
| CRP | X | X | X | X |  | X | X | X | X | X |
| ESR (local) | X | X | X | X |  | X | X | X | X | X |
| QoL (SF-36) assessment | X | X | X | X |  | X | X | X | X | X |
| Hand and foot X-ray | X |  |  |  |  |  |  |  |  |  |
| Safety and other assessments—predose |  |  |  |  |  |  |  |  |  |  |
| Physical examination, vital signs, and weight | X | X | X | X |  | X | X | X | X | X |
| Clinical laboratory tests | X | X | X | X |  | X | X | X | X | X |
| Urine pregnancy test |  | X | X | X |  | X | X | X | X | X |
| 12-lead ECG | X | X |  |  |  |  |  |  |  | X |
| Immunogenicity |  | X | X | X |  | X | X | X | X | X |
| PK blood sampling |  | X | X | X |  | X | X | X | X | X |
| Rheumatoid factor |  | X | X | X |  | X | X | X | X | X |
| Anti-CCP |  | X | X | X |  | X | X | X | X | X |
| Study treatment |  | X | X | X | X | X | X | X | X | X |
| PRE- and POST-SIAQ |  |  |  | X | X | X |  |  |  | X |
| Self-injection assessment checklist by observer |  |  |  | X | X | X |  |  |  | X |
| Hypersensitivity/allergic reaction monitoring and injection-site reaction |  | X | X | X | X | X | X | X | X | X |
| Local site pain by VAS |  | X | X | X |  | X |  | X |  | X |
| Prior/concomitant medications | X | | | | | | | | | |
| Tuberculosis clinical monitoring | X | | | | | | | | | |
| AEs | X | | | | | | | | | |

*A visit window of ±2 days was allowed, based on the previous dosing date, from Dose 2.

*AE* adverse event, *anti-CCP* anti–cyclic citrullinated peptide, *CRP* C-reactive protein, *ECG* electrocardiogram, *ESR* erythrocyte sedimentation rate, *HIV* human immunodeficiency virus, *IGRA* interferon-γ release assay, *PK* pharmacokinetic, *QoL* quality of life, *SF-36* 36-item Short Form Health Survey, *SIAQ* Self-Injection Assessment Questionnaire, *VAS* Visual Analog Scale

## Analysis populations

The intention-to-treat (ITT) population consisted of all subjects enrolled and randomized to receive a dose of either study drug, regardless of whether or not any study drug dosing was completed.

The per-protocol (PP) population consisted of all randomized subjects who had received all full doses of study drug up to week 22 and had an ACR assessment at week 24. If a subject received all doses of study drug up to week 22 but delayed study drug administration >7 days from the previous dose, before week 24, the subject was excluded from the PP population. Major protocol deviations that could affect the interpretation of results for the primary efficacy endpoint also resulted in the exclusion of a subject from the PP population, as determined at the blinded data review meeting.

The pharmacokinetic (PK) population consisted of all subjects who received ≥1 full dose of either study drug and had ≥1 post-treatment PK concentration result. The PK population was the primary analysis population for PK data.

The safety population consisted of all subjects who received ≥1 full or partial dose of either study drug; this was the primary analysis population for safety data.

The usability population consisted of all subjects in the safety population who self-injected either study drug and had ≥1 evaluable usability measurement (assessed in Bulgaria and Poland only). The usability population was the primary analysis population for usability data.

# SUPPLEMENTARY RESULTS

**Supplementary Table 3.** ACR50 and ACR70 response rates and hybrid ACR scores up to week 24 (ITT population)

|  | CT-P17 (*N* = 324) | EU-adalimumab (*N* = 324) |
| --- | --- | --- |
| ACR50 response, *n* (%) |  |  |
| Week 2 | 15 (4.6) | 15 (4.6) |
| Week 4 | 66 (20.4) | 71 (21.9) |
| Week 8 | 112 (34.6) | 121 (37.3) |
| Week 12 | 151 (46.6) | 155 (47.8) |
| Week 16 | 170 (52.5) | 170 (52.5) |
| Week 20 | 195 (60.2) | 198 (61.1) |
| Week 24 | 195 (60.2) | 206 (63.6) |
| ACR70 response, *n* (%) |  |  |
| Week 2 | 5 (1.5) | 5 (1.5) |
| Week 4 | 20 (6.2) | 21 (6.5) |
| Week 8 | 47 (14.5) | 62 (19.1) |
| Week 12 | 96 (29.6) | 89 (27.5) |
| Week 16 | 99 (30.6) | 108 (33.3) |
| Week 20 | 125 (38.6) | 136 (42.0) |
| Week 24 | 132 (40.7) | 144 (44.4) |
| Hybrid ACR score, mean (SD) |  |  |
| Week 2 | 22.1 (17.9) | 21.1 (18.6) |
| Week 4 | 33.5 (23.1) | 33.8 (24.4) |
| Week 8 | 43.0 (24.5) | 44.1 (25.8) |
| Week 12 | 49.8 (25.8) | 50.3 (26.0) |
| Week 16 | 53.7 (24.8) | 54.1 (26.9) |
| Week 20 | 59.0 (23.9) | 58.2 (26.6) |
| Week 24 | 59.0 (25.2) | 59.8 (26.3) |

*ACR* American College of Rheumatology, *ACR##* ##% improvement according to American College of Rheumatology criteria, *EU-adalimumab* European Union-approved adalimumab, *ITT* intention-to-treat, *SD* standard deviation

**Supplementary Table 4.** Mean (SD) C_trough_ overall and by ADA status* (PK population)

| C_trough_ (μg/l), mean (SD) | CT-P17 (*N* = 321) | EU-adalimumab (*N* = 323) |
| --- | --- | --- |
| Week 0 | 2,790.1 (1,093.88) | 2,535.4 (1,019.19) |
| Week 2 | 4,609.9 (2,026.10) | 4,204.1 (1,937.42) |
| Week 6 | 6,476.4 (3,384.95) | 5,955.7 (3,288.61) |
| Week 10 | 7,506.2 (3,922.57) | 6,652.6 (3,855.41) |
| Week 14 | 7,831.7 (4,272.83) | 6,982.4 (4,130.99) |
| Week 18 | 7,979.8 (4,449.91) | 7,093.7 (4,294.13) |
| Week 22 | 8,015.4 (4,560.54) | 7,380.1 (4,504.41) |
| ADA-positive subgroup | *N* = 143 | *N* = 185 |
| Week 0 | 2,512.2 (1,185.87) | 2,366.0 (1,060.25) |
| Week 2 | 3,786.8 (2,124.59) | 3,748.2 (2,041.30) |
| Week 6 | 4,508.0 (3,235.16) | 4,720.9 (3,399.39) |
| Week 10 | 5,233.2 (3,846.36) | 5,140.4 (3,943.34) |
| Week 14 | 5,542.7 (4,074.01) | 5,228.2 (4,111.68) |
| Week 18 | 5,336.6 (4,053.24) | 5,471.8 (4,398.53) |
| Week 22 | 5,299.9 (4,148.01) | 5,700.1 (4,744.96) |
| ADA-negative subgroup | *N* = 178 | *N* = 138 |
| Week 0 | 3,013.4 (960.50) | 2,761.3 (917.77) |
| Week 2 | 5,266.5 (1,680.93) | 4,802.1 (1,613.43) |
| Week 6 | 8,033.3 (2,605.05) | 7,587.2 (2,281.08) |
| Week 10 | 9,332.6 (2,901.80) | 8,668.9 (2,625.18) |
| Week 14 | 9,654.9 (3,484.61) | 9,237.7 (2,878.90) |
| Week 18 | 10,023.6 (3,589.67) | 9,178.9 (3,101.94) |
| Week 22 | 10,083.6 (3,701.30) | 9,524.0 (3,055.90) |

*ADA-positive subgroup: subjects with ≥1 post-treatment ADA-positive result; ADA-negative subgroup: subjects with negative post-treatment ADA results throughout.

*ADA* antidrug antibody, *C_trough_* trough serum concentration, *EU-adalimumab* European Union-approved adalimumab, *PK* pharmacokinetic, *SD* standard deviation

**Supplementary Figure 1.** Mean (±SD) C_trough_ by ADA status (PK population).


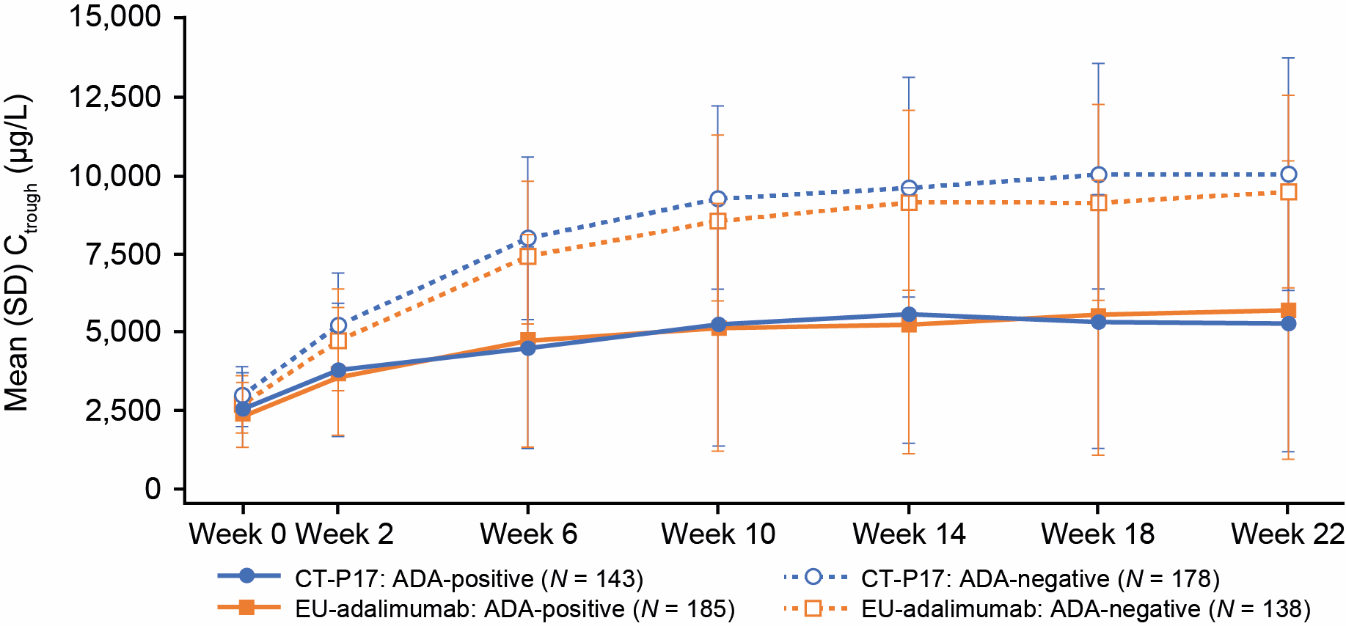


*ADA* antidrug antibody, *C_trough_* trough serum concentration, *EU-adalimumab* European Union-approved adalimumab, *PK* pharmacokinetic, *SD* standard deviation

**Supplementary Table 5.** Mean (SD) scores by domain for PRE-SIAQ and POST-SIAQ (usability population)

| Domain  Visit | SIAQ | CT-P17 (*N* = 70) | | EU-adalimumab (*N* = 76) | |
| --- | --- | --- | --- | --- | --- |
|  |  | *n* | Mean (SD)* | *n* | Mean (SD)* |
| Feelings about injections |  |  |  |  |  |
| Week 4 | PRE | 59 | 7.78 (2.459) | 70 | 8.00 (2.092) |
|  | POST | 59 | 8.21 (2.247) | 70 | 8.15 (2.193) |
| Week 6 | PRE | 66 | 7.97 (2.021) | 72 | 8.11 (1.978) |
|  | POST | 66 | 7.75 (2.143) | 72 | 8.17 (2.011) |
| Week 8 | PRE | 66 | 8.09 (2.045) | 73 | 7.83 (2.234) |
|  | POST | 66 | 7.89 (2.119) | 74 | 8.03 (2.035) |
| Week 24 | PRE | 64 | 8.05 (2.243) | 73 | 8.38 (1.767) |
|  | POST | 64 | 8.15 (2.225) | 73 | 8.26 (1.968) |
| Self-confidence |  |  |  |  |  |
| Week 4 | PRE | 59 | 6.02 (2.311) | 70 | 5.52 (2.086) |
|  | POST | 59 | 6.61 (2.417) | 70 | 5.85 (2.277) |
| Week 6 | PRE | 66 | 6.39 (2.022) | 72 | 6.12 (2.243) |
|  | POST | 66 | 6.70 (1.974) | 72 | 6.27 (2.353) |
| Week 8 | PRE | 66 | 6.60 (2.100) | 72 | 6.55 (1.709) |
|  | POST | 66 | 6.72 (2.158) | 74 | 6.41 (2.087) |
| Week 24 | PRE | 64 | 6.84 (2.064) | 73 | 6.87 (1.992) |
|  | POST | 64 | 6.90 (1.821) | 73 | 6.94 (1.925) |
| Self-image |  |  |  |  |  |
| Week 4 | POST | 59 | 8.69 (2.244) | 70 | 8.00 (2.842) |
| Week 6 | POST | 66 | 8.45 (2.311) | 72 | 7.74 (2.558) |
| Week 8 | POST | 66 | 8.45 (2.548) | 74 | 7.84 (2.389) |
| Week 24 | POST | 64 | 8.13 (2.635) | 73 | 8.42 (2.104) |
| Pain and skin reactions during or after the injection | | | | | |
| Week 4 | POST | 59 | 9.42 (1.096) | 70 | 9.50 (1.138) |
| Week 6 | POST | 66 | 9.45 (1.155) | 72 | 9.59 (0.888) |
| Week 8 | POST | 66 | 9.70 (0.756) | 74 | 9.40 (1.138) |
| Week 24 | POST | 64 | 9.40 (1.199) | 73 | 9.49 (1.194) |
| Ease of use of the self-injection device | | | | | |
| Week 4 | POST | 59 | 8.15 (1.754) | 70 | 7.98 (1.388) |
| Week 6 | POST | 66 | 8.01 (1.778) | 72 | 8.09 (1.272) |
| Week 8 | POST | 66 | 8.16 (1.592) | 74 | 7.99 (1.430) |
| Week 24 | POST | 64 | 8.14 (1.664) | 73 | 8.30 (1.680) |
| Satisfaction with self-injection |  |  |  |  |  |
| Week 4 | PRE | 59 | 7.88 (1.904) | 70 | 7.93 (1.902) |
|  | POST | 59 | 7.58 (1.475) | 70 | 7.26 (1.351) |
| Week 6 | PRE | 66 | 8.14 (1.659) | 72 | 7.95 (1.588) |
|  | POST | 66 | 7.59 (1.355) | 72 | 7.38 (1.254) |
| Week 8 | PRE | 66 | 7.99 (1.766) | 72 | 7.78 (1.707) |
|  | POST | 66 | 7.66 (1.381) | 74 | 7.38 (1.341) |
| Week 24 | PRE | 64 | 7.89 (2.146) | 73 | 7.84 (1.924) |
|  | POST | 64 | 7.87 (1.333) | 73 | 7.91 (1.255) |

*Domain scores ranged from 0 (worst) to 10 (best) and was defined as the mean of transformed item scores included in the domain. For items on a 5-point semantic Likert-type scale, transformed scores were calculated as (raw score − 1) × 2.5; for items on a 6-point semantic Likert-type scale, transformed scores were calculated as (raw score − 1) × 2. Domain scores were only calculated if at least half of the questions were completed for that domain.

*EU-adalimumab* European Union-approved adalimumab, *SD* standard deviation, *SIAQ* Self-injection Assessment Questionnaire

**Supplementary Table 6.** Study drug–related TESAEs (safety population)

| Subjects, n (%) | CT-P17 (*N* = 324) | EU-adalimumab (*N* = 324) |
| --- | --- | --- |
| Study drug–related TESAEs | 5 (1.5) | 4 (1.2) |
| Neutropenia—grade 4 | 1 (0.3) | 1 (0.3) |
| Abdominal pain—grade 2 | 1 (0.3) | 0 |
| Acute kidney injury—grade 4 | 1 (0.3) | 0 |
| Cellulitis—grade 3 | 1 (0.3) | 0 |
| Erysipelas—grade 3 | 1 (0.3) | 0 |
| Gastroenteritis rotavirus—grade 4 | 1 (0.3) | 0 |
| Hepatic failure—grade 4 | 1 (0.3) | 0 |
| Hypertension—grade 3 | 0 | 1 (0.3) |
| Pyelonephritis acute—grade 3 | 0 | 1 (0.3) |
| Tuberculosis—grade 3 | 0 | 1 (0.3) |

*EU-adalimumab* European Union-approved adalimumab, *TESAE* treatment-emergent serious adverse event

**Supplementary Table 7.** TEAEs by System Organ Class reported by ≥2% of subjects in either treatment group (safety population)

| System Organ Class, n (%) | CT-P17 (*N* = 324) | EU-adalimumab (*N* = 324) |
| --- | --- | --- |
| Blood and lymphatic system disorders | 24 (7.4) | 24 (7.4) |
| Gastrointestinal disorders | 24 (7.4) | 16 (4.9) |
| General disorders and administration site conditions | 25 (7.7) | 30 (9.3) |
| Infections and infestations | 97 (29.9) | 103 (31.8) |
| Injury, poisoning, and procedural complications | 8 (2.5) | 5 (1.5) |
| Investigations | 32 (9.9) | 27 (8.3) |
| Metabolism and nutrition disorders | 12 (3.7) | 6 (1.9) |
| Musculoskeletal and connective tissue disorders | 13 (4.0) | 15 (4.6) |
| Nervous system disorders | 19 (5.9) | 9 (2.8) |
| Renal and urinary disorders | 8 (2.5) | 4 (1.2) |
| Skin and subcutaneous tissue disorders | 10 (3.1) | 14 (4.3) |
| Vascular disorders | 6 (1.9) | 7 (2.2) |

*EU-adalimumab* European Union-approved adalimumab, *TEAE* treatment-emergent adverse event

**Supplementary Table 8.** Summary of immunogenicity results (safety population)

| Subjects, n (%) | CT-P17 (*N* = 324) | EU-adalimumab (*N* = 324) |
| --- | --- | --- |
| Week 0 |  |  |
| ADA-positive | 11 (3.4) | 6 (1.9) |
| NAb-positive | 4 (1.2) | 1 (0.3) |
| ADA-negative | 313 (96.6) | 318 (98.1) |
| Week 2 |  |  |
| ADA-positive | 43 (13.3) | 86 (26.5) |
| NAb-positive | 15 (4.6) | 21 (6.5) |
| ADA-negative | 278 (85.8) | 236 (72.8) |
| Week 4 |  |  |
| ADA-positive | 79 (24.4) | 108 (33.3) |
| NAb-positive | 35 (10.8) | 40 (12.3) |
| ADA-negative | 242 (74.7) | 214 (66.0) |
| Week 8 |  |  |
| ADA-positive | 80 (24.7) | 98 (30.2) |
| NAb-positive | 59 (18.2) | 67 (20.7) |
| ADA-negative | 239 (73.8) | 224 (69.1) |
| Week 12 |  |  |
| ADA-positive | 88 (27.2) | 116 (35.8) |
| NAb-positive | 72 (22.2) | 97 (29.9) |
| ADA-negative | 228 (70.4) | 202 (62.3) |
| Week 16 |  |  |
| ADA-positive | 91 (28.1) | 121 (37.3) |
| NAb-positive | 85 (26.2) | 104 (32.1) |
| ADA-negative | 222 (68.5) | 195 (60.2) |
| Week 20 |  |  |
| ADA-positive | 96 (29.6) | 112 (34.6) |
| NAb-positive | 92 (28.4) | 105 (32.4) |
| ADA-negative | 215 (66.4) | 202 (62.3) |
| Week 24 |  |  |
| ADA-positive | 93 (28.7) | 116 (35.8) |
| NAb-positive | 83 (25.6) | 103 (31.8) |
| ADA-negative | 216 (66.7) | 196 (60.5) |

*ADA* antidrug antibody, *EU-adalimumab* European Union-approved adalimumab, *NAb* neutralizing antibody

**Supplementary Table 9.** ACR20 response rate at week 24 by ADA status* (ITT population)

|  | CT-P17 (*N* = 324) | EU-adalimumab (*N* = 324) |
| --- | --- | --- |
| ADA-positive subgroup, n/N (%) | 119/143 (83.2) | 148/185 (80.0) |
| ADA-negative subgroup, n/N (%) | 149/178 (83.7) | 120/138 (87.0) |

*ADA-positive subgroup: subjects with ≥1 post-treatment ADA-positive result; ADA-negative subgroup: subjects with negative post-treatment ADA results throughout

*ACR20* 20% improvement by American College of Rheumatology criteria, *ADA* antidrug antibody, *EU-adalimumab* European Union-approved adalimumab, *ITT* intention-to-treat

**Supplementary Table 10.** Treatment-emergent adverse events by ADA status (safety population)

| Subjects, n/N (%) | CT-P17 (*N* = 324) | EU-adalimumab  (*N* = 324) |
| --- | --- | --- |
| ≥1 TEAE |  |  |
| ADA-positive | 83/143 (58.0) | 113/185 (61.1) |
| ADA-negative | 86/178 (48.3) | 71/138 (51.4) |
| ≥1 TESAE |  |  |
| ADA-positive | 6/143 (4.2) | 8/185 (4.3) |
| ADA-negative | 4/178 (2.2) | 8/138 (5.8) |
| ≥1 TEAE classified as hypersensitivity/allergic reactions |  |  |
| ADA-positive | 0 | 4/185 (2.2) |
| ADA-negative | 2/178 (1.1) | 0 |
| ≥1 TEAE classified as ISR |  |  |
| ADA-positive | 8/143 (5.6) | 14/185 (7.6) |
| ADA-negative | 8/178 (4.5) | 8/138 (5.8) |

*ADA* antidrug antibody, *EU-adalimumab* European Union-approved adalimumab, *ISR* injection-site reaction, *TEAE* treatment-emergent adverse event, *TESAE* treatment-emergent serious adverse event

# SUPPLEMENTARY MATERIALS FOR DISCUSSION

**Supplementary Figure 2.** Historical data for ACR20 response rate at week 24^a^ for reference or biosimilar adalimumab (50 mg/ml), compared with CT-P17 or reference adalimumab (100 mg/ml) treatment in the current study (ITT population) [3-7]


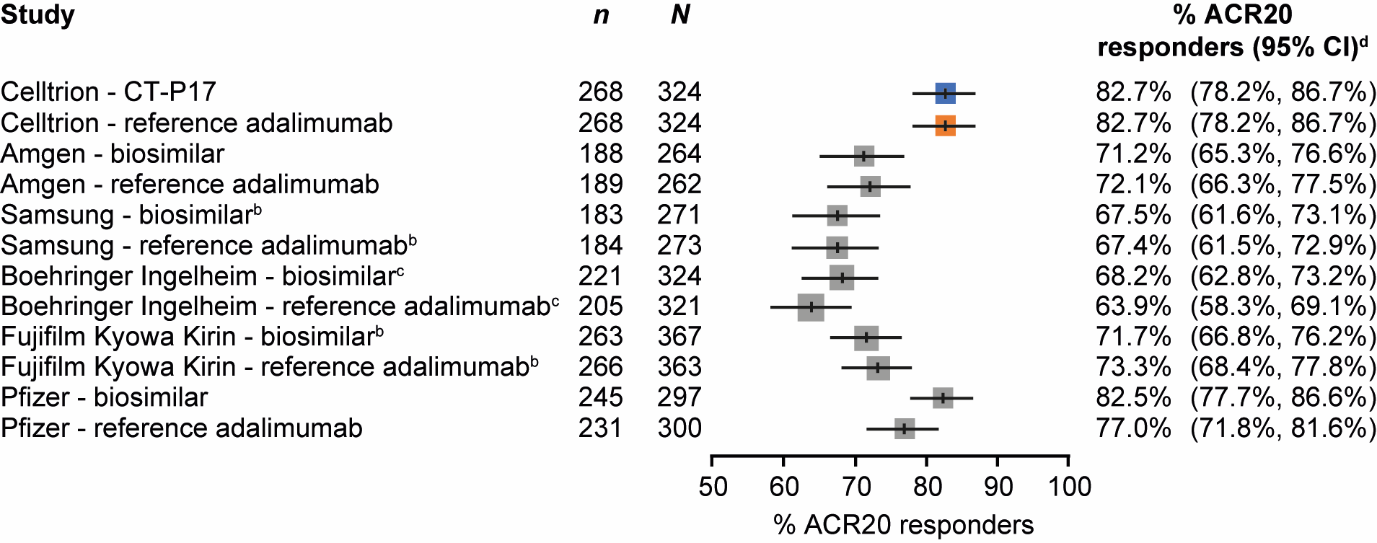


^a^For Pfizer, the result is at week 26.

^b^n is based on non-responder imputation in the full analysis set; N is for the ITT population.

^c^n is estimated using ACR20 response rate and N; ACR20 response rate displayed is adjusted for the n calculated in this meta-analysis of historical data.

^d^95% CIs were calculated based on n/N and ACR20 response rates.

*ACR20* 20% improvement by American College of Rheumatology criteria, *CI* confidence interval, *ITT* intent-to-treat, *n* number of subjects who achieved ACR20 at week 24, *N* number of subjects in the ITT population

# REFERENCES FOR SUPPLEMENTARY MATERIAL

1. Aletaha D, Neogi T, Silman AJ*, et al.* 2010 rheumatoid arthritis classification criteria: an American College of Rheumatology/European League Against Rheumatism collaborative initiative. Ann Rheum Dis 2010;69:1580-8.

2. Hochberg MC, Chang RW, Dwosh I*, et al.* The American College of Rheumatology 1991 revised criteria for the classification of global functional status in rheumatoid arthritis. Arthritis Rheum 1992;35:498-502.

3. US Food and Drug Administration Center for Drug Evaluation and Research. Statistical review and evaluation: ABP 501. 2016; Available at: <https://www.accessdata.fda.gov/drugsatfda_docs/nda/2016/761024Orig1s000StatR.pdf>. Accessed July 7, 2020.

4. US Food and Drug Administration Center for Drug Evaluation and Research. Biosimilar multi-disciplinary evaluation and review: SB5. 2019; Available at: <https://www.accessdata.fda.gov/drugsatfda_docs/nda/2019/761059Orig1s000MultidisciplineR.pdf>. Accessed July 7, 2020.

5. US Food and Drug Administration Center for Drug Evaluation and Research. Statstical review and evaluation: BI 695501. 2017; Available at: <https://www.accessdata.fda.gov/drugsatfda_docs/nda/2017/761058Orig1s000StatR.pdf>. Accessed July 7, 2020.

6. European Medicines Agency. Assessment report: Hulio. 2018; Available at: <https://www.ema.europa.eu/en/documents/assessment-report/hulio-epar-public-assessment-report_en.pdf>. Accessed July 7, 2020.

7. US Food and Drug Administration Center for Drug Evaluation and Research. Biosimilar multi-disciplinary evaluation and review: PF-06410293. 2019; Available at: <https://www.accessdata.fda.gov/drugsatfda_docs/nda/2019/761118Orig1s000MultidisciplineR.pdf>. Accessed May 28, 2020.

1. *Co-senior authors. †Address correspondence to Professor Edward Keystone, Rebecca MacDonald Centre, Mount Sinai Hospital, University of Toronto, Toronto, Canada; edkeystone@rheumkey.com [↑](#footnote-ref-1)
